# Giant calvarial solitary fibrous tumor with intracranial and extracranial extension in an elderly patient: imaging and surgical considerations

**DOI:** 10.1016/j.radcr.2026.05.057

**Published:** 2026-06-16

**Authors:** James A. Sumawe

**Affiliations:** a Department of Radiology, Mbeya Zonal Referral Hospital, Mbeya, Tanzania; b University of Dar es Salaam–Mbeya College of Health and Allied Sciences, Mbeya, Tanzania

**Keywords:** Solitary fibrous tumor, Calvarial tumor, Intracranial and extracranial extension

## Abstract

Solitary fibrous tumors (SFTs) are rare hypervascular mesenchymal tumors that may occasionally involve the calvarium with both intracranial and extracranial extension. We report the case of an 87-year-old hypertensive woman who presented with progressive right-sided weakness and worsening headache associated with a large left parietal scalp mass. Contrast-enhanced CT and MRI demonstrated a giant left parietal calvarial lesion with heterogeneous enhancement and extensive intra- and extracranial components causing mass effect with midline shift. Preoperative imaging suggested a hypervascular skull lesion, and surgical en bloc resection was performed with removal of involved bone. Histopathological examination confirmed a spindle-cell neoplasm with staghorn vascular pattern, nuclear atypia, and bone involvement, consistent with solitary fibrous tumor. The patient had an uneventful postoperative recovery with clinical improvement. This case highlights the importance of imaging in characterizing calvarial masses and guiding surgical planning and management.

## Introduction

Solitary fibrous tumors (SFTs) are uncommon mesenchymal neoplasms of fibroblastic origin, now recognized as a unified entity encompassing lesions previously termed hemangiopericytomas, following advances in molecular characterization and the identification of NAB2–STAT6 gene fusions that underpin their shared pathogenesis. According to the World Health Organization (WHO) Classification of Central Nervous System Tumors, SFTs are classified as a spectrum of fibroblastic tumors with variable biological behavior, ranging from benign to highly aggressive forms with recurrence and metastatic potential, leading to the retirement of the term “hemangiopericytoma” in modern nomenclature [1,2].

Intracranial SFTs are rare, accounting for approximately 1% of all primary central nervous system tumors, and typically present as extra-axial, dural-based masses that radiologically mimic meningiomas due to overlapping imaging features such as avid contrast enhancement and well-circumscribed margins [3,4]. However, SFTs tend to demonstrate more aggressive clinical behavior, with a significant propensity for local recurrence and distant metastasis even many years after initial resection, necessitating long-term surveillance [5,6].

Calvarial involvement with transosseous extension into both intracranial and extracranial compartments is exceedingly rare and represents an unusual and diagnostically challenging manifestation of SFT. Such cases may demonstrate extensive bone destruction, marked vascularity, and mass effect, often leading to diagnostic confusion with other hypervascular skull lesions, including meningioma, metastasis, or primary bone sarcomas [7,8]. Recent literature highlights that imaging modalities such as contrast-enhanced MRI and CT play a critical role in preoperative characterization, particularly in identifying hypervascularity, bone invasion, and the full extent of tumor spread, which directly influence surgical planning and the risk of intraoperative hemorrhage [9,10].

The present case is noteworthy due to its extreme rarity in terms of giant size, extensive intra- and extracranial calvarial involvement, and presentation in an elderly patient, which collectively pose significant diagnostic and therapeutic challenges. This highlights the importance of considering SFT in the differential diagnosis of aggressive calvarial masses, particularly when imaging demonstrates a highly vascular lesion with bone destruction and dual compartment extension.

## Case presentation

### Patient information

An 87-year-old female with a history of poorly controlled hypertension presented with progressive neurological and local symptoms. She reported a 1-month history of progressively worsening right-sided weakness and a 1-week history of increasing headache, associated with a large, progressively enlarging left parietal scalp mass.

### Clinical findings

On admission, the patient was fully conscious (GCS 15/15) but appeared neurologically compromised, with dense right-sided hemiparesis involving both the upper and lower limbs with motor power graded at 1/5. A large, fixed, nontender left parietal scalp mass was noted. Oxygen saturation was 88% on room air, and blood pressure variability was consistent with poorly controlled hypertension. No signs of meningeal irritation were present, and general examination was otherwise unremarkable.

### Imaging findings

Contrast-enhanced CT and MRI of the brain demonstrated a large expansile left parietal calvarial lesion measuring approximately 8 × 8 × 9 cm. The lesion showed heterogeneous postcontrast enhancement, internal cystic and necrotic areas, and prominent intratumoral vascular channels (Fig. 1). There was extensive intra- and extracranial extension with significant mass effect, resulting in compression of the left lateral ventricle and a rightward midline shift of approximately 6.4 mm (Figs. 2 and 4). The lesion also abutted and compressed the superior sagittal sinus and was associated with surrounding vasogenic edema (Fig. 3). Overall imaging features were suggestive of a highly vascular aggressive calvarial tumor.

**Fig. 1:**
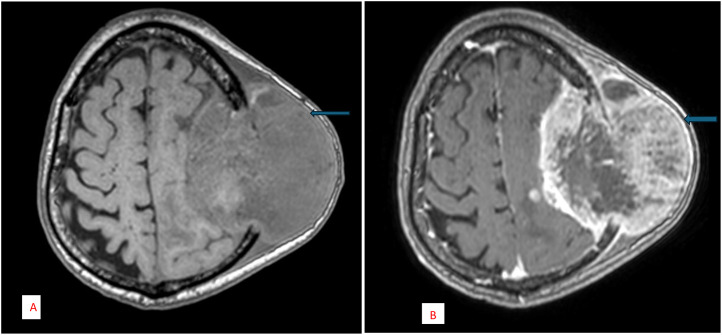
Axial T1 precontrast (A) and postcontrast (B) MRI showing a large left parietal expansile bone lesion measuring approximately 8 × 8 × 9 cm with heterogeneous enhancement, internal fluid areas, and prominent internal vascular channels. Note the soft tissue components with both intra- and extracranial extension.

**Fig. 2:**
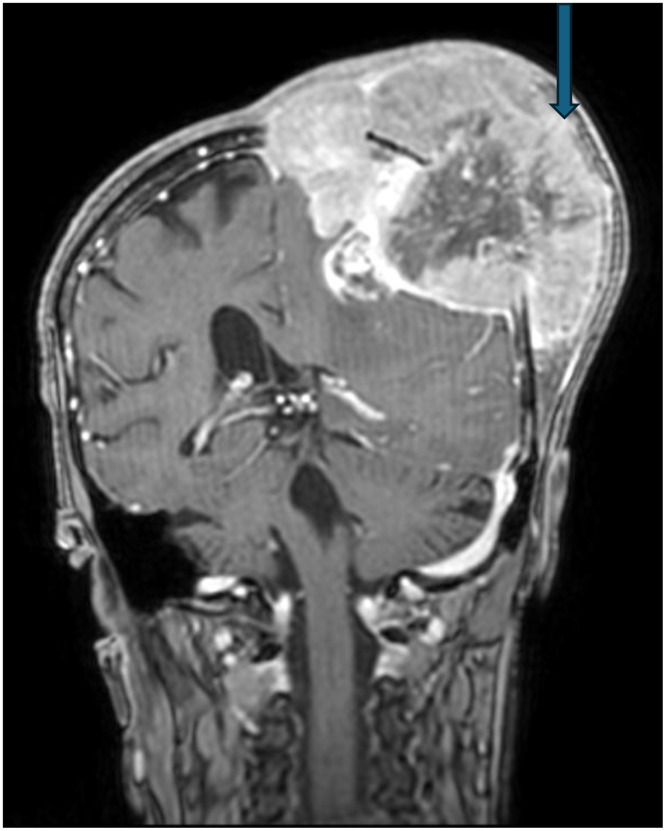
Coronal T1 postcontrast MRI demonstrating compression of the left lateral ventricle and rightward midline shift of 6.4 mm. The lesion abuts and compresses the anterior superior sagittal sinus. Surrounding vasogenic edema is visible.

**Fig. 3:**
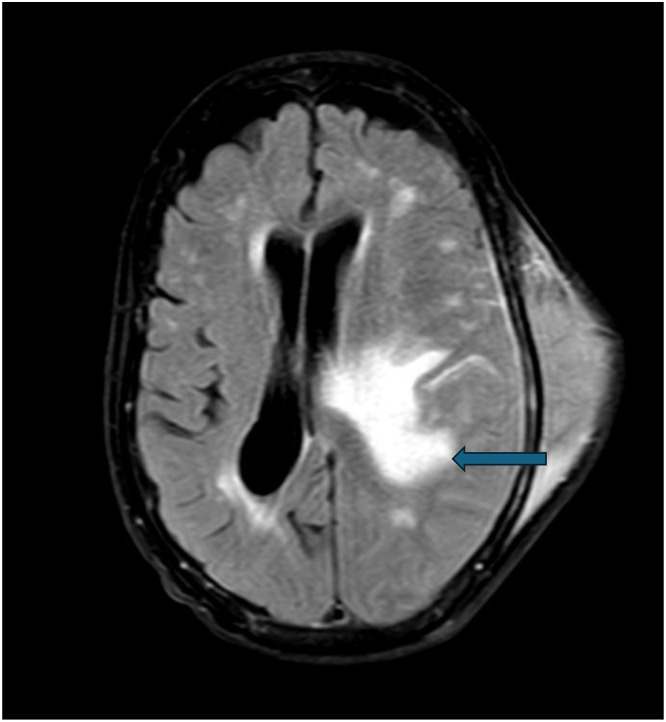
Axial FLAIR sequence MRI image showing periventricular bright halo and bilateral deep and subcortical hyperintense foci, consistent with chronic small vessel ischemic changes and reactive edema adjacent to the tumor.

**Fig. 4:**
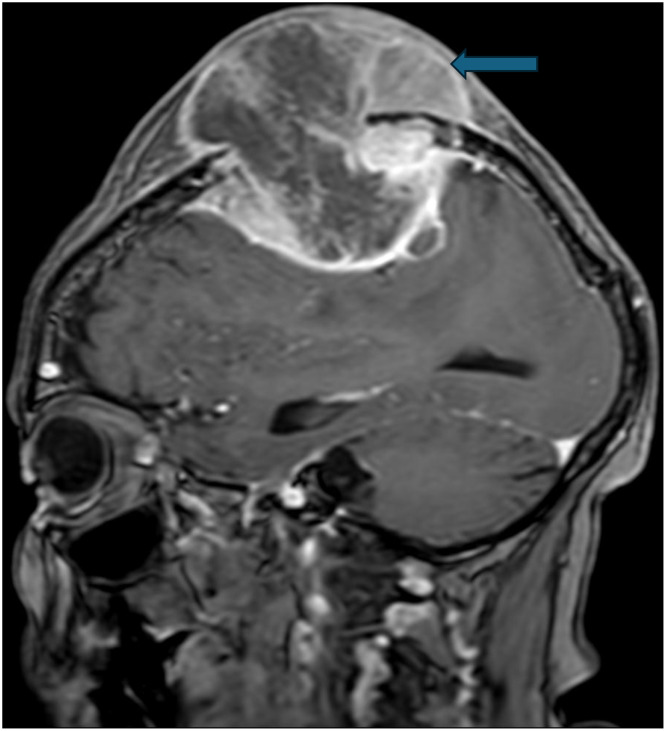
Sagittal T1 postcontrast MRI illustrating the intra- and extracranial extent of the lesion, marked mass effect on adjacent brain parenchyma, and displacement of surrounding structures.

*Differential diagnoses* included a solitary fibrous tumor/hemangiopericytoma spectrum lesion, plasmacytoma, and primary calvarial osteosarcoma.

Preoperative metastatic workup, including contrast-enhanced CT of the chest and abdomen, was performed and did not reveal evidence of distant metastatic disease.

### Therapeutic intervention

Given the imaging findings of a highly vascular calvarial tumor with significant mass effect, the patient underwent elective surgical resection. Preoperative angiographic embolization was considered given the highly vascular nature of the lesion; however, it was not performed due to limited institutional resources and logistical constraints. Nevertheless, meticulous intraoperative vascular control was achieved through proximal carotid exposure and careful surgical hemostasis. A combined neurosurgical and vascular control approach was adopted. Right cervical exposure was performed to achieve carotid and external carotid artery control, followed by a horseshoe scalp incision with wide exposure. Multiple burr holes were created, and a craniotomy extending across the midline was performed. En bloc resection of the tumor along with the involved calvarial bone was achieved. No intradural extension was identified intraoperatively. Hemostasis was secured using Surgicel and bone wax, and cranioplasty was performed using mesh and microscrew fixation. A surgical drain was placed. The estimated intraoperative blood loss was approximately 800 mL. The patient was subsequently transferred to the intensive care unit postoperatively.

### Histopathological findings

Histopathological examination of the resected specimen revealed a highly cellular spindle and ovoid cell neoplasm arranged in a patternless architecture with characteristic staghorn-like vascular channels. There was marked nuclear atypia and increased mitotic activity, averaging approximately 5 per high-power field. Tumor infiltration into bone was identified, with positive surgical margins in sampled areas. Overall morphological features were consistent with a solitary fibrous tumor (SFT).

Immunohistochemistry, including STAT6, CD34, and SMA, was recommended for confirmation; however, it was not performed due to unavailability of the tests at the institution and financial constraints faced by the patient.

Regarding pathology documentation, frozen section analysis and high-resolution scanned histology images were not available due to lack of a dedicated scanning facility. Only routine light microscopy was performed. Nevertheless, the diagnosis was established based on characteristic histomorphological features in correlation with radiological and intraoperative findings.

### Postoperative course and follow-up

The immediate postoperative course was initially stable, with improvement in neurological status and uneventful recovery in the intensive care unit. The patient was successfully extubated and transferred to the ward with stable vital signs.

During follow-up, recurrent scalp swelling was noted over the surgical site and supra-auricular region, accompanied by recurrence of right-sided weakness. Imaging follow-up was advised urgently due to suspicion of tumor recurrence. Long-term management was planned with multidisciplinary tumor board discussion, repeat imaging, and consideration of re-operation depending on disease progression.

## Discussion

Solitary fibrous tumors (SFTs) are uncommon mesenchymal neoplasms characterized by a spectrum of biological behavior ranging from indolent growth to locally aggressive and metastatic disease. The contemporary understanding of SFT has evolved significantly following the identification of the NAB2–STAT6 fusion gene, which underpins its unified pathogenesis and supports its distinction from other spindle cell neoplasms previously grouped under hemangiopericytoma terminology [1,3]. According to the 2021 WHO Classification of Central Nervous System Tumors, SFT and hemangiopericytoma are now considered a single entity with a shared molecular basis and variable clinical aggressiveness [1].

Calvarial SFTs with simultaneous intra- and extracranial extension remain exceptionally rare, with only few isolated cases described in the literature. These lesions may present as large, destructive, hypervascular skull masses with significant mass effect, often mimicking more common entities such as plasmacytoma, metastasis, or primary bone sarcomas [4,9]. In the present case, the extensive bone destruction, marked vascularity, and dual compartment extension initially supported a broad differential diagnosis, reflecting the well-recognized diagnostic overlap on conventional imaging [11].

Radiologically, SFTs typically demonstrate avid heterogeneous enhancement, flow voids or internal vascular channels, and aggressive osseous remodeling when calvarial involvement occurs. However, imaging alone is not specific, and histopathological correlation remains essential. The present case demonstrated classic morphological features, including a patternless architecture of spindle to ovoid cells, prominent staghorn-like vascular channels, and moderate mitotic activity, all of which are strongly supportive of SFT in the appropriate clinical and radiological context [3,12].

Although STAT6 immunohistochemistry is considered the diagnostic gold standard, its absence does not preclude a confident diagnosis when characteristic histomorphological features are present. Multiple recent studies and WHO-aligned reviews emphasize that the combination of classic morphology (patternless spindle cell proliferation, staghorn vasculature, and collagenous stroma) with concordant radiological findings is sufficient for diagnosis in resource-limited settings, particularly when molecular or immunohistochemical testing is unavailable [1,3,13]. In this case, the diagnosis was further supported by intraoperative findings of a highly vascular tumor arising from the calvarium with bone infiltration and absence of features typical of plasmacytoma (eg, sheets of plasma cells) or osteosarcoma (eg, malignant osteoid production). Therefore, despite the absence of STAT6 confirmation, the integrated clinico-radiological-pathological correlation strongly supports SFT as the most consistent diagnosis.

Surgical management remains the mainstay of treatment, with gross total or en bloc resection associated with improved local control and reduced recurrence risk. In highly vascular calvarial tumors, preoperative planning with vascular control is critical to minimize intraoperative blood loss and neurological morbidity. In the present case, carotid control and meticulous hemostasis were essential given the tumor’s marked vascularity and proximity to major venous sinuses. These principles are strongly supported in recent neurosurgical literature on skull-base and calvarial SFTs, which emphasize the importance of achieving maximal safe resection in anatomically complex regions [11,13].

Despite complete macroscopic resection, SFTs are well known for late recurrence and potential malignant progression, even in cases initially considered histologically benign. Factors associated with aggressive behavior include high mitotic index, necrosis, and positive surgical margins, all of which were present in this case to varying degrees. This underscores the necessity for long-term surveillance with serial imaging, as recurrence may occur many years after initial treatment [5,12].

In summary, this case highlights a rare calvarial SFT with extensive intra- and extracranial involvement in an elderly patient. It also emphasizes that, in resource-limited environments, a definitive diagnosis can still be reliably established through integrated clinico-radiological and histomorphological correlation even in the absence of STAT6 immunohistochemistry, provided that classical diagnostic features are present and alternative diagnoses are reasonably excluded.

## Conclusion

Giant calvarial solitary fibrous tumors with intra- and extracranial extension are rare lesions that may closely mimic other hypervascular skull tumors. Accurate preoperative imaging assessment and meticulous surgical planning are essential to guide safe resection. Despite complete excision, these tumors require long-term surveillance due to their potential for recurrence.

## Informed consent

Written informed consent was obtained from the patient’s legal guardian for publication of clinical details and images.

## Author contributions

James Alex Sumawe: Concept, patient care, manuscript drafting, data collection, manuscript review.

## Patient consent

Patient consent was obtained from the patient’s relatives for publication of this case report and any accompanying images. The patient’s identity has been protected, and no identifying information is included in the manuscript.
